# Finger-Ring Test and Low Muscle Mass in Older Adults with Chronic Kidney Disease: A Cross-Sectional Study

**DOI:** 10.3390/medicina62081447

**Published:** 2026-07-25

**Authors:** Büşragül Yılmaz, Serap Boz, Fatma Kaplan Efe, Rıdvan Erten, Ertuğrul Demirel, Hande Selvi Öztorun, Rana Tuna Doğrul, Meryem Keleş, Hemrin Kavak, Büşra Betül Çağır, Gunes Eken, Fatih Dede, Kamile Sılay

**Affiliations:** 1 Department of Geriatrics, Ankara Bilkent City Hospital, Ankara 06800, Türkiye; serapsen85@hotmail.com (S.B.); drefe.106@gmail.com (F.K.E.); dr.ridvanerten@gmail.com (R.E.); drertugruldemirel@gmail.com (E.D.); rana_tuna@hotmail.com (R.T.D.); hemrinkavak@gmail.com (H.K.); drbbetul@outlook.com (B.B.Ç.); 2 Division of Geriatrics, Department of Internal Medicine, Faculty of Medicine, Ankara Yıldırım Beyazıt University, Ankara 06800, Türkiye; drhandeslv@hotmail.com (H.S.Ö.); kamilesilay@hotmail.com (K.S.); 3 Department of Nephrology, Ankara Bilkent City Hospital, Ankara 06800, Türkiye; meryem7980@gmail.com (M.K.); fatded@yahoo.com (F.D.); 4 Department of Geriatrics, University of Health Sciences, Ankara Bilkent City Hospital, Ankara 06800, Türkiye; drgunesarik@gmail.com

**Keywords:** chronic kidney disease, sarcopenia, low muscle mass, finger-ring test, Yubi-wakka, older adults

## Abstract

*Background and Objectives*: Sarcopenia is highly prevalent among older adults with chronic kidney disease (CKD) and is associated with adverse clinical outcomes. The finger-ring (Yubi-wakka) test is a simple anthropometric screening tool based on calf circumference; however, its performance in older adults with CKD remains unclear. This study aimed to investigate the association of the finger-ring test with low muscle mass, sarcopenia, and comprehensive geriatric assessment parameters and to evaluate its diagnostic performance for identifying low muscle mass in older adults with CKD. *Materials and Methods*: This cross-sectional study included 115 patients aged ≥65 years with CKD who were evaluated in geriatric and nephrology inpatient services. After excluding two participants with missing finger-ring measurements, 113 individuals were analyzed. Muscle mass was assessed using bioelectrical impedance analysis and low muscle mass was defined according to EWGSOP2-based Turkish cut-off values. Demographic characteristics, anthropometric measurements, laboratory findings, and comprehensive geriatric assessment parameters were recorded. Correlation analyses, logistic regression models, receiver operating characteristic (ROC) analyses, and likelihood-ratio tests were performed. *Results*: Among 113 participants, 62 were classified as finger-ring positive (FR = 0) and 51 as finger-ring negative (FR = 1). Low muscle mass was significantly more frequent in the FR = 0 group than in the FR = 1 group (51.6% vs. 24.0%, *p* = 0.005). Finger-ring test results showed strong correlations with calf circumference (rho = 0.689, *p* < 0.001) and body mass index (BMI) (rho = 0.631, *p* < 0.001), whereas the correlation with muscle mass was modest (rho = 0.250, *p* = 0.008). For detecting low muscle mass, the finger-ring test demonstrated an area under the curve (AUC) of 0.643 (95% CI 0.554–0.732), sensitivity of 72.7%, specificity of 55.9%, positive predictive value of 51.6%, and negative predictive value of 76.0%. In multivariable analyses, BMI remained the strongest independent determinant of finger-ring test results, whereas the association with muscle mass lost statistical significance after BMI adjustment. Adding age and sex significantly improved discrimination, while the contribution of nutritional status was limited. *Conclusions*: The finger-ring test is associated with low muscle mass in older adults with CKD; however, its diagnostic performance is modest and appears to be substantially influenced by body size and calf circumference. Therefore, the finger-ring test should be considered a simple adjunctive screening tool rather than a stand-alone method for identifying low muscle mass in this population.

## 1. Introduction

Chronic kidney disease (CKD) is a highly burdensome clinical condition that is closely associated with functional decline, malnutrition, frailty, and adverse clinical outcomes in older adults. The uremic milieu, chronic inflammation, metabolic acidosis, hormonal dysregulation, physical inactivity, and inadequate nutritional intake may disrupt muscle protein turnover, leading to reductions in both muscle mass and muscle strength [1,2,3]. Consequently, sarcopenia is of particular importance in older adults with CKD, not only from a geriatric assessment perspective but also for prognostic evaluation and care planning [2,3].

According to the updated European Working Group on Sarcopenia in Older People (EWGSOP2) consensus, sarcopenia is defined as a progressive muscle disease characterized primarily by low muscle strength, confirmed by low muscle quantity or quality, and classified as severe when accompanied by impaired physical performance [4]. Recent systematic reviews and meta-analyses conducted in CKD populations have demonstrated that sarcopenia is highly prevalent and that loss of muscle strength affects a substantial proportion of patients [2]. However, direct assessment of muscle mass is not always readily available in routine clinical practice. Therefore, there remains a need for simple, rapid, low-cost, bedside screening tools that can be easily implemented in everyday clinical settings [3,4].

Anthropometric approaches based on calf circumference have attracted considerable attention in this regard. The EWGSOP2 consensus recommends calf circumference as a practical surrogate marker in settings where advanced body composition assessment methods are unavailable [4]. The finger-ring (Yubi-wakka) test, which compares calf circumference with a ring formed by the thumbs and index fingers, represents one of the simplest applications of this approach. Population-based studies have reported associations between the finger-ring test and sarcopenia, functional decline, and mortality risk, while more recent validation studies have shown promising, albeit variable, diagnostic performance [5,6,7].

Nevertheless, most of the available evidence regarding the finger-ring test has been derived from community-dwelling older adults. In contrast, hospitalized older adults with CKD represent a clinically distinct and more vulnerable population, characterized by a higher burden of multimorbidity, frailty, chronic inflammation, protein-energy wasting, physical inactivity, and functional impairment. In addition, factors such as fluid retention, peripheral edema, alterations in body composition, and malnutrition may influence calf circumference measurements and consequently reduce the diagnostic accuracy of calf circumference–based screening methods in this population [3,8]. Therefore, these disease-specific characteristics may influence the performance of the finger-ring test itself. Consequently, findings from community-based populations cannot be directly extrapolated to hospitalized older adults with CKD. Validation of the finger-ring test in this specific clinical setting is therefore essential to determine whether this simple bedside screening tool retains its clinical utility and diagnostic performance in routine geriatric and nephrology practice.

## 2. Materials and Methods

### 2.1. Study Design and Participants

This cross-sectional observational study was based on prospectively collected data and was conducted to investigate the association of the finger-ring test with muscle mass and comprehensive geriatric assessment parameters in older adults with chronic kidney disease (CKD). A total of 115 patients evaluated in the Geriatrics and Nephrology inpatient services of Ankara Bilkent City Hospital between July 2022 and March 2024 were included. The study was approved by the Clinical Research Ethics Committee (Approval Date: 21 December 2021; Approval No: E1-21-2168). The study was conducted in accordance with the principles of the Declaration of Helsinki. Written informed consent was obtained from all participants.

#### 2.1.1. Participant Selection

Individuals aged 65 years and older with a diagnosis of CKD were eligible for inclusion. Because muscle mass was assessed using bioelectrical impedance analysis (BIA), participants receiving maintenance hemodialysis were evaluated only immediately after their dialysis session, when fluid balance was considered most stable, in order to minimize the influence of hydration status on body composition measurements. Participants not receiving maintenance hemodialysis underwent BIA during the comprehensive geriatric assessment performed on the same clinical evaluation. However, the number and distribution of patients receiving maintenance hemodialysis were not retained as separate variables in the analytical dataset. Therefore, these data could not be retrieved, and subgroup analyses according to dialysis modality could not be performed. This standardized approach was adopted to improve the accuracy of BIA measurements.

#### 2.1.2. Clinical and Geriatric Assessment

Demographic characteristics, anthropometric measurements, laboratory parameters, and comprehensive geriatric assessment findings were obtained from patient records. Age, sex, educational status, body mass index (BMI), right and left calf circumference, serum albumin, estimated glomerular filtration rate (eGFR), dominant handgrip strength, and gait speed were recorded. Estimated glomerular filtration rate (eGFR) was calculated using the 2021 CKD-EPI creatinine equation, and chronic kidney disease stage was classified according to the Kidney Disease: Improving Global Outcomes (KDIGO) categories (G3a–G5).

Comprehensive geriatric assessment included the Katz Activities of Daily Living (ADL) Index, Lawton Instrumental Activities of Daily Living (IADL) Scale, Mini Nutritional Assessment–Short Form (MNA-SF), Geriatric Depression Scale–Short Form (GDS-SF), FRAIL Scale, Fried frailty phenotype, SARC-F questionnaire, number of medications, polypharmacy status, history of falls, osteoporosis, and dynapenia.

#### 2.1.3. Finger-Ring Test Procedure and Classification

The finger-ring (Yubi-wakka) test was the primary variable of interest in this study. Participants were instructed to identify the widest circumference of the calf on their non-dominant leg. A ring was then formed using the thumbs and index fingers of both hands and placed around the calf at its widest point. The test was performed twice, and the consistent result was recorded.

The test was categorized into three levels: 0 = calf circumference smaller than the finger ring (a gap remained between the ring and the calf); 1 = calf circumference exactly matched the finger ring; and 2 = calf circumference larger than the finger ring (the fingers could not completely encircle the calf). For analytical purposes, categories 0 and 1 were combined and defined as a positive test result (FR = 0), whereas category 2 was defined as a negative test result (FR = 1).

Two participants with missing finger-ring test measurements were excluded from the analyses. For diagnostic performance analyses aimed at identifying low muscle mass, FR = 0 was considered a positive test result [5].

#### 2.1.4. Inclusion Criteria

- Age ≥ 65 years
- Diagnosis of chronic kidney disease (CKD)
- Ability to cooperate with study assessments and procedures
- Provision of written informed consent

#### 2.1.5. Exclusion Criteria

- Advanced dementia
- Active delirium
- Severe visual or hearing impairment that could interfere with participation in study assessments
- Neuromuscular disorders (e.g., amyotrophic lateral sclerosis, myopathy, peripheral neuropathy)
- Major acute illness (e.g., acute myocardial infarction, acute stroke, or sepsis)
- Metastatic cancer with an expected survival of less than 3 months
- Severe edema that could substantially affect anthropometric or bioelectrical impedance measurements. Severe edema was assessed during physical examination performed by the study investigators. Participants with clinically evident bilateral pitting edema were excluded because edema was considered likely to influence the accuracy of anthropometric and bioelectrical impedance measurements.

### 2.2. Muscle Mass Assessment and Definition of Sarcopenia

Muscle mass was assessed using bioelectrical impedance analysis (BIA). Body composition and skeletal muscle mass were measured using a multifrequency bioelectrical impedance analyzer (Bodystat QuadScan 4000, Bodystat Ltd., Isle of Man, UK). In participants receiving maintenance hemodialysis, BIA measurements were performed immediately after the hemodialysis session. In participants not receiving maintenance hemodialysis, BIA was performed during the comprehensive geriatric assessment after at least 5 min of rest in the supine position and under clinically stable conditions without overt volume overload. Individuals with permanent cardiac pacemakers or metallic implants were excluded from BIA assessment.

Low muscle mass was defined according to the recommendations of the European Working Group on Sarcopenia in Older People 2 (EWGSOP2) using sex-specific cut-off values for the skeletal muscle mass index (SMMI) derived from BIA and validated in the Turkish population: <9.2 kg/m^2^ for men and <7.4 kg/m^2^ for women [9].

Handgrip strength was measured using a digital hand dynamometer (Grip-D T.K.K. 5401, Takei Scientific Instruments Co., Ltd., Niigata, Japan) according to the recommendations of the American Society of Hand Therapists (ASHT) [10]. Participants were assessed in a seated position with the shoulder adducted and neutrally rotated, the elbow flexed at 90°, the forearm in a neutral position, and the wrist positioned between 0° and 30° of extension and between 0° and 15° of ulnar deviation. Three consecutive measurements were obtained from the dominant hand, and the highest value was used for the analyses. Low muscle strength was defined as handgrip strength <32 kg in men and <22 kg in women, based on Turkish population-specific cut-off values [9]. Participants with low muscle strength according to these cut-off values were classified as having dynapenia.

Sarcopenia was classified according to the EWGSOP2 algorithm using measures of muscle strength, muscle mass, and physical performance. Low physical performance was defined as a gait speed of ≤0.8 m/s in accordance with EWGSOP2 recommendations. Following the EWGSOP2 diagnostic framework, low muscle mass and sarcopenia variables were not included simultaneously in the same multivariable model.

### 2.3. Statistical Analysis

Data quality was assessed before statistical analyses. An outlying value in the education variable was recoded as missing data, and an implausible value identified in the FRAIL scale was considered a data-entry error and excluded from the analyses. Missing data rates were compared between finger-ring test groups, and no significant differences were detected in evaluable comparisons. Complete-case analysis was applied for derived binary categorical variables, and participants with missing data in the source variable were excluded from the corresponding analyses. Missing data were infrequent for the core analytical variables (fewer than 2% of values; only one participant lacked a muscle-mass measurement); complete-case analysis was therefore considered equivalent to a full-data analysis, and multiple imputation was not performed because it was unlikely to change the estimates. Variables with extensive missingness that were not used in the core analyses, such as LDL cholesterol, were excluded from those analyses.

Statistical analyses were performed using Python 3.12 with the SciPy 1.14, Statsmodels 0.14, and Scikit-learn 1.5 packages. Continuous variables were evaluated according to their distributional characteristics. Because departures from normality were common, continuous variables were summarized as medians and interquartile ranges (IQRs), whereas categorical variables were presented as frequencies and percentages. Normality was assessed using the Shapiro–Wilk test.

Given the frequent non-normal distribution of continuous variables and to ensure methodological consistency, between-group comparisons were performed using the Mann–Whitney U test for continuous variables and the chi-square test for categorical variables; Fisher’s exact test was used when expected cell counts were <5. Associations between variables were evaluated using Spearman’s rank correlation analysis.

Binary logistic regression analyses were performed to identify independent determinants of finger-ring test results. Variables associated with the outcome at *p* < 0.10 in univariable analyses were considered candidates for multivariable models. Calf circumference was not included in multivariable models because it represents the anatomical basis of the finger-ring test and was highly correlated with body mass index (BMI).

Model performance was evaluated using the Hosmer–Lemeshow goodness-of-fit test, Nagelkerke pseudo-R^2^, and classification accuracy. The diagnostic performance of the finger-ring test for identifying low muscle mass was assessed using the area under the receiver operating characteristic curve (AUC), sensitivity, specificity, positive predictive value, negative predictive value, likelihood ratios, and the Youden index. Confidence intervals for diagnostic accuracy measures were calculated using the Wilson method. Additional subgroup analyses were performed according to BMI categories. In addition, finger-ring test results and low muscle mass were examined across KDIGO CKD stages (G3a–G5), and a finger-ring × CKD stage interaction term was tested to assess whether the association varied by stage.

The incremental contribution of variables added to nested models was evaluated using likelihood-ratio tests. Therefore, the DeLong test was not used to compare AUC values between nested models [11]. The discriminative performance of the final model was internally validated using 1000 bootstrap resamples with optimism correction. For the multivariable logistic regression models, the number of events per variable was calculated to assess overfitting; with 51 outcome events, it ranged from 12.8 to 17.0 across models, exceeding the recommended minimum of 10. On the basis of the available sample size, the study had approximately 80% power to detect an area under the curve of about 0.66 for identifying low muscle mass at a two-sided α of 0.05; subgroup analyses were correspondingly less well powered and were regarded as exploratory. Potential differences in discriminative performance across BMI categories were evaluated using a finger-ring × BMI interaction term. Because of multiple comparisons, borderline findings were considered exploratory. All statistical tests were two-sided, and a *p*-value < 0.05 was considered statistically significant.

## 3. Results

**Figure 1:**
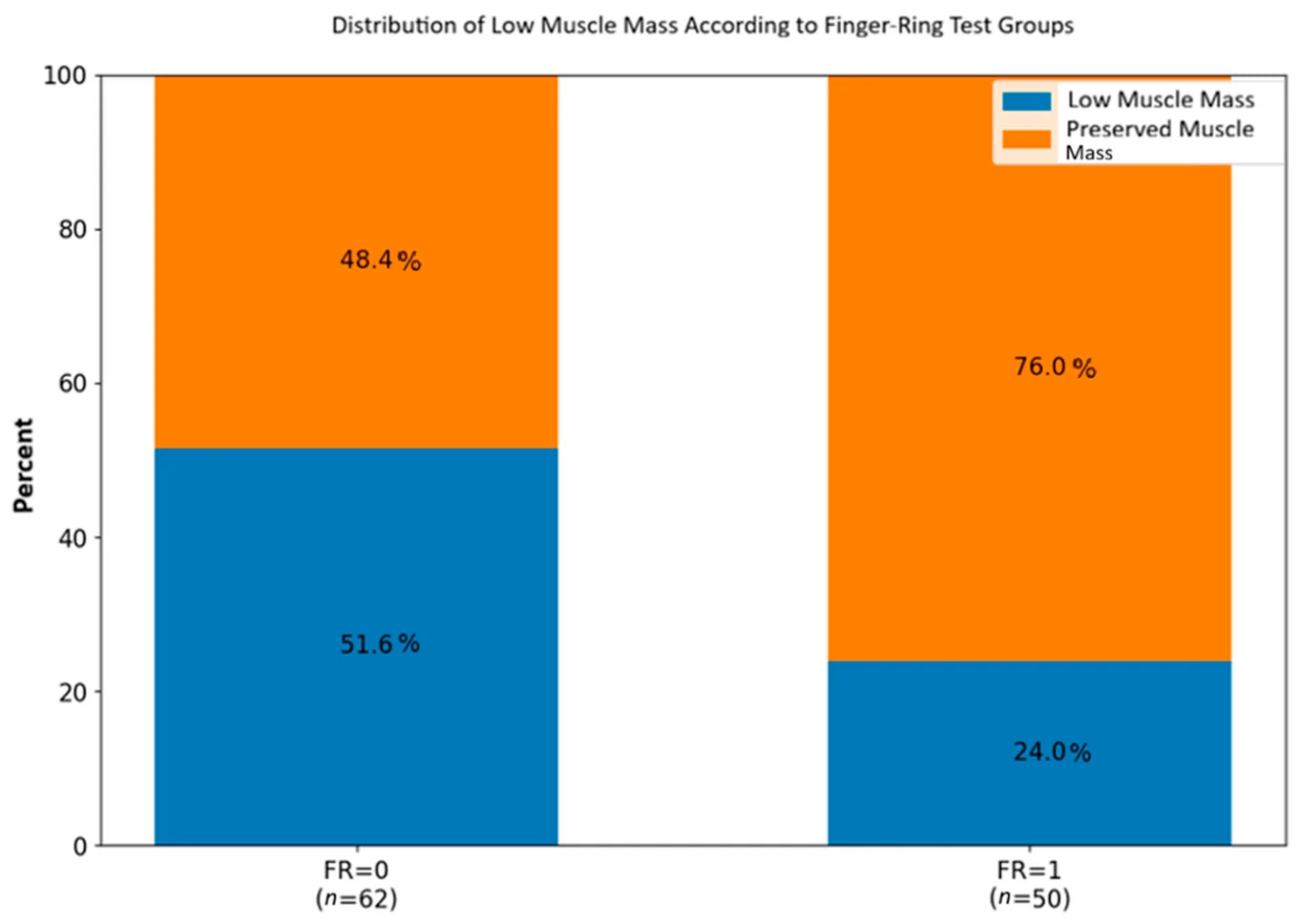
Distribution of Low and Preserved Muscle Mass According to Finger-Ring Test Groups. Muscle mass data were unavailable for one participant in the FR = 1 group; therefore, this individual was excluded from the figure (*n* = 62 for FR = 0 and *n* = 50 for FR = 1).

A total of 115 patients were enrolled in the study. After being recorded in three categories, finger-ring test results were dichotomized (FR = 0: calf circumference smaller than or equal to the finger ring; FR = 1: calf circumference larger than the finger ring). Two participants with missing finger-ring measurements were excluded, leaving 113 individuals for analysis, including 62 in the FR = 0 group and 51 in the FR = 1 group. The participant selection process is shown in Figure 1. In the missing data analysis, no significant differences in the proportion of missing observations were observed between the finger-ring test groups in evaluable comparisons.

The demographic, anthropometric, and laboratory characteristics of the participants are summarized in Table 1. Comparison of demographic and clinical characteristics showed that age distribution was similar between the two groups (median 74 [69–80] vs. 73 [69–77] years, *p* = 0.554). Female sex was more common in the FR = 1 group than in the FR = 0 group (28/51 [54.9%] vs. 19/62 [30.6%], *p* = 0.016). BMI was significantly higher in the FR = 1 group (31.6 [28.1–35.4] vs. 24.6 [22.2–27.3] kg/m^2^, *p* < 0.001). Low muscle mass was more prevalent in the FR = 0 group (32/62 [51.6%] vs. 12/50 [24.0%], *p* = 0.005). Both right and left calf circumferences were significantly greater in the FR = 1 group (both *p* < 0.001). In contrast, no significant differences were observed between the groups with respect to eGFR, serum albumin, sex-specific dominant handgrip strength, or gait speed.

According to KDIGO categories, the cohort comprised predominantly moderate-to-advanced CKD: 21 participants (18.6%) were classified as stage G3a, 22 (19.5%) as G3b, 31 (27.4%) as G4, and 39 (34.5%) as G5, with none in the G1–G2 range (median eGFR 22 mL/min/1.73 m^2^). The distribution of CKD stages did not differ significantly between the finger-ring test groups (*p* = 0.113). The association between finger-ring positivity and low muscle mass persisted after adjustment for CKD stage (adjusted odds ratio 3.11, 95% CI 1.35–7.14, *p* = 0.008), and the finger-ring × CKD stage interaction was not significant (*p* = 0.765), indicating a consistent association across CKD stages.

The comprehensive geriatric assessment findings are summarized in Table 2. When comprehensive geriatric assessment parameters were examined, the MNA-SF score was significantly higher in the FR = 1 group (12 [10–13] vs. 11 [8–12], *p* = 0.019). Malnutrition risk, defined according to the MNA-SF, was more frequent in the FR = 0 group (38/60 [63.3%] vs. 20/51 [39.2%], *p* = 0.019). The number of medications did not differ significantly between groups (7 [5–9] vs. 8 [6–11], *p* = 0.075). The distribution of sarcopenia categories differed nominally between groups (*p* = 0.039), with confirmed and severe sarcopenia being more common in the FR = 0 group; however, this finding should be interpreted as exploratory because of multiple comparisons and small cell counts. No significant differences were observed in Katz ADL, Lawton IADL, GDS-SF, FRAIL score, SARC-F score, polypharmacy, or history of falls. Although dynapenia was more frequent in the FR = 0 group (95.2% vs. 84.3%), the difference did not reach statistical significance (*p* = 0.063).

**Figure 2:**
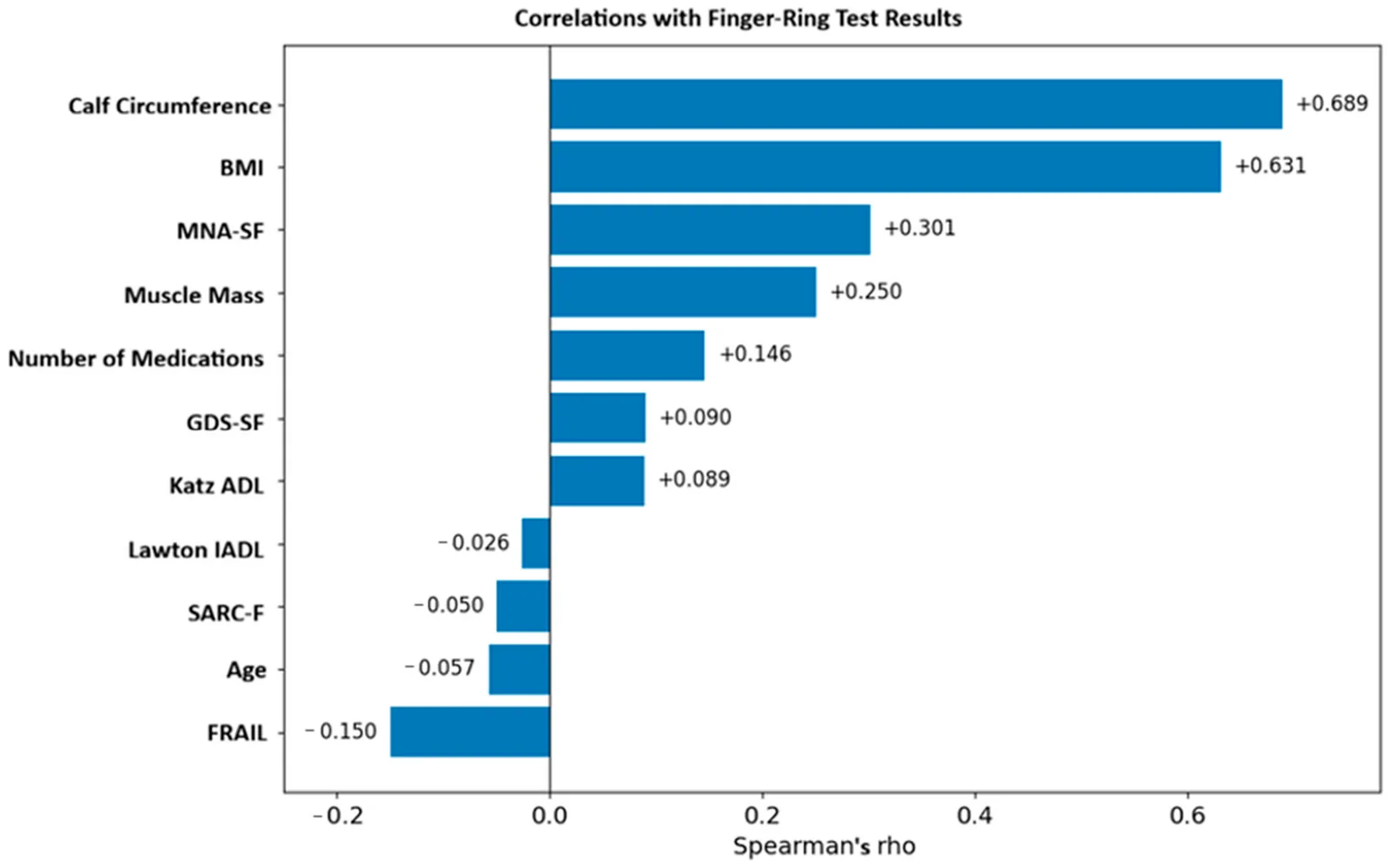
Spearman Correlation Coefficients Between Finger-Ring Test Results and Selected Clinical Variables. Significant positive correlations were observed between finger-ring test results and calf circumference, body mass index (BMI), MNA-SF score, and muscle mass (all *p* < 0.05). No significant correlations were found for the remaining variables.

The Spearman correlation coefficients between the finger-ring test results and the evaluated variables are illustrated in Figure 2. In Spearman correlation analyses, the strongest associations with finger-ring test results were observed for calf circumference (rho = 0.689, *p* < 0.001) and BMI (rho = 0.631, *p* < 0.001). A weak-to-moderate positive correlation was found between finger-ring test results and muscle mass (rho = 0.250, *p* = 0.008). MNA-SF scores also showed a weak-to-moderate positive correlation (rho = 0.301, *p* = 0.001). No significant correlations were identified with number of medications, GDS-SF, FRAIL score, age, SARC-F score, Katz ADL, Lawton IADL, handgrip strength, or gait speed.

The diagnostic performance of the finger-ring test for identifying low muscle mass is summarized in Table 3. In the diagnostic performance analysis for low muscle mass (complete-case analysis, *n* = 112), the finger-ring test yielded an AUC of 0.643 (95% CI: 0.554–0.732). Sensitivity was 72.7% (95% CI: 58.2–83.7), specificity was 55.9% (95% CI: 44.1–67.1), positive predictive value was 51.6% (95% CI: 39.4–63.6), and negative predictive value was 76.0% (95% CI: 62.6–85.7). The positive likelihood ratio (LR+) was 1.65 (95% CI: 1.19–2.28), the negative likelihood ratio (LR−) was 0.49 (95% CI: 0.29–0.83), overall accuracy was 62.5%, and the Youden index was 0.286. Because these likelihood ratios produced only limited changes in post-test probability, the finger-ring test cannot be considered a stand-alone diagnostic tool. According to BMI categories, the AUC values were 0.536, 0.575, and 0.662, respectively; however, differences in discriminative performance were not statistically significant in the formal interaction analysis (*p* = 0.44). As BMI increased, sensitivity decreased (90% to 40%), whereas specificity increased (17% to 92%). The high specificity observed in the obese subgroup suggests that increasing body size may shift classification errors toward false-negative results; therefore, this finding should be interpreted as exploratory.

The results of the univariable and multivariable logistic regression analyses are presented in Table 4. In univariable logistic regression analyses, right calf circumference (OR 1.64, 95% CI: 1.36–1.98, *p* < 0.001), BMI (OR 1.44, 95% CI: 1.26–1.64, *p* < 0.001), preserved muscle mass (OR 3.12, 95% CI: 1.40–6.96, *p* = 0.006), MNA-SF score (OR 1.23, 95% CI: 1.05–1.44, *p* = 0.009), male sex (OR 0.36, 95% CI: 0.17–0.79, *p* = 0.010), and number of medications (OR 1.16, 95% CI: 1.01–1.32, *p* = 0.031) were significantly associated with finger-ring test results. In multivariable models, BMI remained the strongest independent predictor across all models (OR range: 1.41–1.45; all *p* < 0.001). In contrast, the association between preserved muscle mass and finger-ring test results weakened after adjustment for BMI and lost statistical significance (adjusted OR range: 2.1–2.7; *p* = 0.08–0.15). Similarly, MNA-SF score, number of medications, and sex were no longer significant after BMI was included in the models. This pattern suggests that finger-ring test results primarily reflect body size, as represented by BMI, and that the observed association with muscle mass is largely mediated through body size.

**Figure 3:**
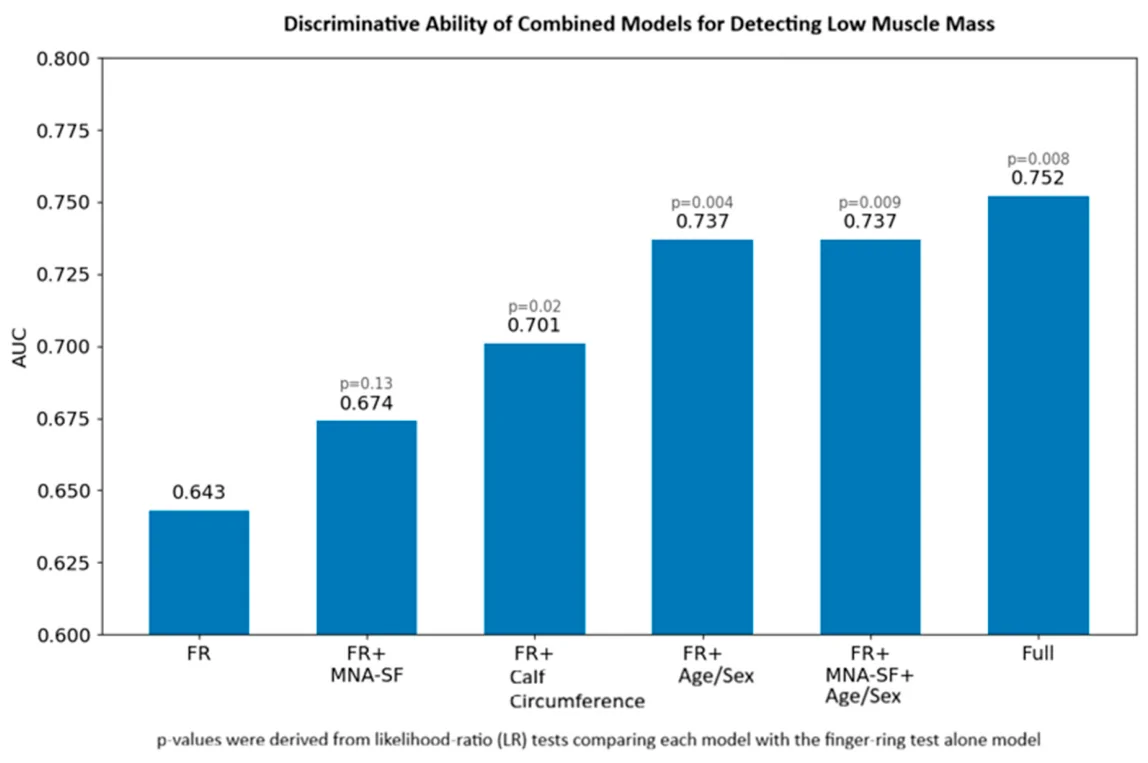
Comparison of the Discriminative Performance of Combined Models for Identifying Low Muscle Mass. Area under the receiver operating characteristic curve (AUC) values are shown for the finger-ring test alone and for combined models. *p*-values were obtained from likelihood-ratio (LR) tests comparing each model with the finger-ring test alone model.

The discriminative performance of the combined models is summarized in Table 5, and the corresponding AUC comparisons are illustrated in Figure 3. In combined model analyses, the finger-ring test alone yielded an AUC of 0.643. The addition of MNA-SF did not significantly improve discrimination (AUC 0.674; likelihood-ratio test *p* = 0.13). In contrast, the addition of calf circumference (AUC 0.701; *p* = 0.019), age and sex (AUC 0.737; *p* = 0.004), and the full model (AUC 0.752; *p* = 0.008) significantly improved discriminative performance compared with the finger-ring test alone. However, because calf circumference represents the direct anatomical basis of the finger-ring test, the observed improvement likely reflects a structural component of the measurement rather than an independent clinical contribution. The finger-ring test provided a statistically significant but modest incremental contribution beyond age and sex (*p* = 0.001), whereas no significant additional contribution was observed after BMI was included in the model (*p* = 0.14). The apparent AUC of the full model was 0.752, and the optimism-corrected bootstrap AUC was 0.71.

## 4. Discussion

In the present study, the finger-ring test was significantly associated with muscle mass in older adults with chronic kidney disease (CKD); however, this association was strongly influenced by overall body size and calf circumference. When used alone, the test showed only modest discriminative ability for identifying low muscle mass (AUC = 0.643), supporting its use as a screening rather than a diagnostic tool. Compared with findings from community-dwelling older populations [5,6], its diagnostic performance appears more limited in older adults with CKD and substantial chronic disease burden. Similarly, a recent systematic review and meta-analysis confirmed the association between the finger-ring test and sarcopenia but highlighted the limited evidence available from clinical populations [12]. Therefore, in this patient population, the finger-ring test may be best considered a practical adjunct to comprehensive clinical assessment rather than a stand-alone screening or diagnostic method.

Our findings indicate that the finger-ring test is primarily associated with anthropometric measures rather than muscle mass alone. The strongest correlations were observed with calf circumference (rho = 0.689) and BMI (rho = 0.631), while BMI remained an independent predictor in all multivariable models. Similarly, Rosa et al. reported a significant association between finger-ring test categories and appendicular lean soft tissue mass measured by dual-energy X-ray absorptiometry (DXA), although this relationship was strongly influenced by body size [13]. Piodena-Aportadera et al. reported that, among community-dwelling older adults, the finger-ring test showed substantially lower diagnostic performance than calf circumference for both low muscle mass (AUC 0.591 vs. 0.855–0.870) and sarcopenia (AUC 0.581 vs. 0.788–0.818) [14]. In our study, the association between the finger-ring test and muscle mass lost statistical significance after adjustment for BMI, suggesting that body size substantially influences test performance. Consistent with this finding, Fujii et al. identified metabolic syndrome as an independent predictor of a positive finger-ring test result among women [15]. Furthermore, the recent Asia–Oceania consensus on sarcopenic obesity recommends cautious interpretation of the finger-ring test in individuals with obesity, acknowledging the potential influence of body habitus on test performance [16].

The weak association between the finger-ring test and functional parameters is also noteworthy. No significant relationships were observed with handgrip strength, gait speed, Katz ADL, Lawton IADL, or frailty measures. Although dynapenia was more frequent in the FR = 0 group, the difference was not statistically significant. These findings suggest that the finger-ring test primarily reflects muscle mass and body size rather than the functional components of sarcopenia defined by the EWGSOP2 framework [4]. Similar findings have been reported in previous studies, in which the finger-ring test was associated with muscle mass but showed limited correlation with objective physical performance measures [14,17]. Furthermore, Lin et al. demonstrated that although the finger-ring test was associated with sarcopenia, calf circumference provided superior diagnostic accuracy [18]. Therefore, the finger-ring test may be best considered a simple preliminary screening tool when direct anthropometric assessment is not feasible, rather than a standalone screening method within the EWGSOP2 diagnostic algorithm.

In the present study, low muscle mass and muscle strength were classified according to the EWGSOP2 diagnostic framework using cut-off values validated for the Turkish population. This approach is consistent with the EWGSOP2 recommendation to apply population-specific reference values whenever validated thresholds are available and may improve the clinical relevance of sarcopenia assessment in the local population. However, because population-specific cut-off values differ across countries, direct comparisons of prevalence estimates and the diagnostic performance of the finger-ring test with studies using other reference thresholds should be interpreted with caution.

The observed association with nutritional status should also be interpreted cautiously. Higher MNA-SF scores and a lower prevalence of malnutrition risk in the FR = 1 group may initially suggest that the finger-ring test reflects nutritional status. However, this association weakened after adjustment for BMI, indicating that it is largely influenced by body size and composition rather than nutritional status alone. Similarly, Li et al. demonstrated that combining the finger-ring test with the SARC-F questionnaire improved screening performance when calf circumference was incorporated [19]. Given the high prevalence of protein-energy wasting in CKD [20], the close relationship among nutritional status, body size, and muscle mass is expected. Therefore, the finger-ring test should not be considered an independent screening tool for malnutrition.

These findings should be interpreted within the specific context of chronic kidney disease. Fluid retention, edema, chronic inflammation, physical inactivity, and protein-energy wasting are common in CKD and may influence both calf circumference and BIA-derived muscle mass measurements [1,3]. Kang et al. demonstrated that volume status directly affects sarcopenia assessment in patients with non-dialysis CKD [8], while Huang et al. identified male sex and older age as independent risk factors for more advanced sarcopenia stages in pre-dialysis CKD [21]. Consistent with these observations, sex was associated with finger-ring test results in our study, although this association weakened after adjustment for BMI, suggesting that body size partly explains this relationship. Importantly, additional analyses showed that the association between the finger-ring test and low muscle mass remained statistically significant after adjustment for CKD stage, and no significant interaction between finger-ring test results and CKD stage was observed. These findings indicate that the observed association was consistent across CKD stages despite the predominance of advanced disease in our cohort. Therefore, the association between the finger-ring test and low muscle mass may reflect not only muscle loss but also alterations in body composition and fluid status. Clinicians should therefore be aware that, particularly in patients prone to edema, the finger-ring test may yield both false-negative and false-positive results, requiring cautious interpretation. Consequently, objective assessment of muscle mass remains essential whenever confirmation of low muscle mass is clinically required.

### Strengths of the Study

This study has several important strengths. It evaluated the finger-ring test in older adults with chronic kidney disease, a clinically relevant but understudied population. In addition, the analysis incorporated multiple domains of comprehensive geriatric assessment, including muscle mass, nutritional status, frailty, depression, functional status, and medication burden, providing a broader clinical perspective. Methodologically, missing data and potential collinearity were carefully addressed during model development. Furthermore, internal validation using bootstrap resampling strengthened the robustness of the predictive models and reduced the risk of overly optimistic performance estimates. Finally, BMI-stratified analyses offered additional insight into the influence of body size on test performance, although no statistically significant interaction was observed.

## 5. Limitations

Several limitations should be acknowledged. First, the cross-sectional design precludes causal inference regarding the associations between the finger-ring test, muscle mass, and geriatric syndromes. Second, this was a single-center study with a relatively modest sample size, which may limit the generalizability of the findings to other populations of older adults with CKD. The limited sample size may also have reduced the statistical power of subgroup analyses and increased the risk of model overfitting; therefore, these findings should be considered exploratory and require confirmation in larger prospective studies.

Although BIA is a practical and clinically feasible method for assessing muscle mass, its accuracy may be influenced by hydration status, particularly in patients with CKD. In our center, BIA measurements were performed immediately after hemodialysis in patients receiving maintenance hemodialysis to minimize the effects of fluid overload. However, hydration status may have varied among non-dialysis patients. In addition, dialysis modality was not available as a separate analytical variable in the study dataset, and objective markers of hydration or volume status were not consistently available. Therefore, adjustment for these factors was not possible. These issues may have affected the accuracy of BIA-derived muscle mass measurements and should be considered when interpreting our findings. Similarly, edema may have influenced both finger-ring test results and calf circumference measurements.

Finally, although internal validation using bootstrap resampling was performed, external validation in independent cohorts is warranted to confirm the robustness and generalizability of the predictive models.

### Clinical Implications

Taken together, these findings suggest that the finger-ring test may provide a useful alert for the presence of low muscle mass in older adults with chronic kidney disease; however, it should not be used as a stand-alone decision-making tool. Although the test showed a higher negative predictive value than positive predictive value in this study, predictive values are influenced by disease prevalence and should therefore be interpreted with caution. Accordingly, a negative finger-ring test may reduce the likelihood of low muscle mass but should not be considered sufficient to exclude the condition, whereas a positive result should always be interpreted in conjunction with further clinical and objective assessments. As BMI increases, the sensitivity of the test decreases while specificity increases; however, differences in discriminative performance across BMI categories were not statistically significant in formal interaction analyses. Therefore, subgroup findings should not be interpreted as evidence of superior diagnostic performance in specific BMI categories but rather as reflecting body size–dependent measurement behavior. Accordingly, interpretation of finger-ring test results should always be considered within the broader context of body composition.

A recent review encompassing more than 50 validated sarcopenia screening tools emphasized that selecting an appropriate instrument according to the clinical setting is of critical importance [22]. In clinical practice, the finger-ring test may be most useful when combined with calf circumference measurement, assessment of muscle strength, nutritional evaluation, and, whenever feasible, objective measurement of muscle mass. Accordingly, a positive finger-ring test should prompt further evaluation within the EWGSOP2 diagnostic framework, including handgrip strength assessment and objective measurement of muscle mass whenever feasible. Conversely, a negative test result may reduce the likelihood of low muscle mass but should not preclude further assessment in patients with persistent clinical suspicion or in those with conditions that may affect test performance, such as obesity or fluid retention. Given that the Asian Working Group for Sarcopenia (AWGS) 2019 consensus also recommends calf circumference and SARC-F as practical screening tools [23], the finger-ring test may be considered a complementary approach, particularly in settings where access to standard measurement instruments is limited.

From a practical perspective, the finger-ring test may be considered as an initial bedside screening tool in hospitalized older adults with CKD, particularly in resource-limited settings or when direct assessment of muscle mass is not immediately available. A positive finger-ring test should prompt further evaluation with handgrip strength assessment, nutritional screening, and objective measurement of muscle mass using validated methods such as BIA or DXA whenever feasible. Conversely, a negative test result may reduce the likelihood of low muscle mass but should not preclude further assessment in patients with persistent clinical suspicion, particularly in those with obesity, fluid retention, or peripheral edema.

Accordingly, a pragmatic clinical approach is to use the finger-ring test as an initial screening step within the comprehensive geriatric assessment, followed by confirmatory evaluation of muscle strength and muscle mass in individuals with positive screening results or ongoing clinical suspicion.

In conclusion, the finger-ring test is a simple, feasible, and pragmatic bedside screening tool that may help identify hospitalized older adults with CKD who require further evaluation for low muscle mass. However, because its diagnostic performance is influenced by body size and fluid status, it should be integrated into a comprehensive geriatric assessment and followed by objective assessment of muscle strength and muscle mass whenever clinically indicated.

## 6. Conclusions

In conclusion, the finger-ring test is a simple, feasible, and inexpensive screening tool that may help identify older adults with CKD at risk of low muscle mass. However, given its modest diagnostic performance and susceptibility to body size and hydration status, it should not be used in isolation for diagnostic decision-making. Confirmation with objective muscle mass assessment remains necessary whenever clinically indicated.

## Figures and Tables

**Table 1:** Demographic, Anthropometric, and Laboratory Characteristics of Participants Stratified by Finger-Ring Test Results.

Variable	Total	FR = 0	FR = 1	*p*-Value
Age, median (IQR), years	73 (69–79)	74 (69–80)	73 (69–77)	0.554
Female sex, *n* (%)	47 (41.6)	19 (30.6)	28 (54.9)	0.016
BMI, median (IQR), kg/m^2^	27.3 (23.6–31.2)	24.6 (22.2–27.3)	31.6 (28.1–35.4)	<0.001
Low muscle mass, *n* (%)	44 (39.3)	32 (51.6)	12 (24.0)	0.005
Right calf circumference, median (IQR), cm	35.0 (32.0–37.5)	32.0 (30.0–34.9)	37.0 (35.8–40.0)	<0.001
Left calf circumference, median (IQR), cm	35.0 (32.0–37.9)	32.0 (30.0–35.0)	37.0 (35.5–40.0)	<0.001
eGFR, median (IQR), mL/min/1.73 m^2^	22.0 (11.0–38.0)	28.0 (14.0–43.5)	20.0 (11.0–33.0)	0.381
Serum albumin, median (IQR), g/L	34.5 (30.2–39.0)	34.0 (31.0–38.0)	35.5 (30.0–40.2)	0.335
Fasting glucose, median (IQR), mg/dL	113.0 (93.5–153.0)	109.5 (89.0–136.2)	122.0 (100.0–168.0)	0.119
Dominant handgrip strength (men), median (IQR), kg	23.3 (17.6–28.4)	23.3 (17.6–27.1)	23.6 (17.6–28.9)	0.58
Dominant handgrip strength (women), median (IQR), kg	13.8 (9.0–18.2)	10.4 (8.2–16.1)	14.7 (10.2–18.7)	0.22
Gait speed, median (IQR), m/s	0.81 (0.63–0.82)	0.81 (0.42–0.85)	0.81 (0.81–0.81)	0.423

**Abbreviations:** BMI, body mass index; eGFR, estimated glomerular filtration rate; IQR, interquartile range. **Footnote:** Continuous variables are presented as median (interquartile range [IQR]) and were compared using the Mann–Whitney U test. Categorical variables are presented as *n* (%) and were compared using the chi-square test or Fisher’s exact test, as appropriate. Dominant handgrip strength represents the maximum value of three measurements and is presented separately for men and women. Because of the well-established sex-related differences in handgrip strength and the unequal sex distribution between the finger-ring test groups (with a higher proportion of women in the FR = 1 group), sex-independent comparisons of handgrip strength were not performed. Due to missing data, the sample size was <113 for eGFR and serum albumin. Analyses of muscle mass and calf circumference were based on 62 participants in the FR = 0 group and 50 participants in the FR = 1 group. MNA-SF scores and malnutrition risk were calculated using denominators of 60 and 51 participants, respectively.

**Table 2:** Comparison of Comprehensive Geriatric Assessment Parameters According to Finger-Ring Test Groups.

Parameter	Total	FR = 0	FR = 1	*p*-Value
Katz ADL, median (IQR)	5 (4–6)	5 (4–6)	5 (5–6)	0.781
Lawton IADL, median (IQR)	7 (4–8)	8 (4–8)	7 (5–8)	0.509
MNA-SF, median (IQR)	11 (9–13)	11 (8–12)	12 (10–13)	0.019
Malnutrition risk (MNA-SF ≤ 11), *n* (%)	58/111 (52.3)	38/60 (63.3)	20/51 (39.2)	0.019
GDS-SF, median (IQR)	3 (2–5)	3 (2–4)	4 (2–6)	0.143
Depression (GDS-SF ≥ 5), *n* (%)	33/111 (29.7)	15/60 (25.0)	18/51 (35.3)	0.330
FRAIL score, median (IQR)	2 (1–3)	2 (1–3)	2 (1–3)	0.452
SARC-F score, median (IQR)	4 (1–6)	4 (1–6)	5 (2–6)	0.688
Number of medications, median (IQR)	7 (5–10)	7 (5–9)	8 (6–11)	0.075
Polypharmacy (≥5 medications), *n* (%)	94/112 (83.9)	50/62 (80.6)	44/50 (88.0)	0.427
Sarcopenia distribution, % (None/Probable/Confirmed/Severe)	4.5/56.4/10.0/29.1 (*n* = 110)	3.3/45.9/11.5/39.3 (*n* = 61)	6.1/69.4/8.2/16.3 (*n* = 49)	0.039
History of falls (≥1), *n* (%)	36/112 (32.1)	18/62 (29.0)	18/50 (36.0)	0.561
Dynapenia, *n* (%)	102/113 (90.3)	59/62 (95.2)	43/51 (84.3)	0.063

Categorical variables are presented as *n*/*N* (%) and were compared using the chi-square test or Fisher’s exact test, as appropriate. Due to missing data, denominators varied across variables.

**Table 3:** Diagnostic Performance of the Finger-Ring Test for Identifying Low Muscle Mass.

Diagnostic Metric	Overall Sample	95% CI/Subgroup Results
AUC	0.643	0.554–0.732
Sensitivity	72.7%	58.2–83.7
Specificity	55.9%	44.1–67.1
Positive Predictive Value	51.6%	39.4–63.6
Negative Predictive Value	76.0%	62.6–85.7
LR+	1.65	1.19–2.28
LR−	0.49	0.29–0.83
Accuracy	62.5%	53.3–70.9
Youden Index	0.286	—
BMI < 25 kg/m^2^ (*n* = 39)	AUC 0.536	Sensitivity 90.5%/Specificity 16.7%
BMI 25–30 kg/m^2^ (*n* = 37)	AUC 0.575	Sensitivity 69.2%/Specificity 45.8%
BMI > 30 kg/m^2^ (*n* = 36)	AUC 0.662	Sensitivity 40.0%/Specificity 92.3%

**Footnote:** Analyses were based on complete-case data (*n* = 112); two participants with missing finger-ring measurements and one participant with missing muscle mass data were excluded. Confidence intervals for proportions were calculated using the Wilson method and for likelihood ratios using the method of Simel et al. FR = 0 was considered a positive test result.

**Table 4:** Univariable and Multivariable Logistic Regression Analyses of Factors Associated with a Negative Finger-Ring Test Result (FR = 1).

Model/Variable	OR	95% CI	*p*-Value
Univariable analysis			
Right calf circumference (cm)	1.641	1.360–1.981	<0.001
BMI (kg/m^2^)	1.436	1.255–1.642	<0.001
Preserved muscle mass	3.378	1.490–7.655	0.004
MNA-SF score	1.232	1.053–1.441	0.009
Male sex	0.363	0.168–0.785	0.010
Number of medications	1.156	1.014–1.318	0.031
Multivariable Model 1			
Preserved muscle mass	2.671	0.910–7.840	0.074
BMI (kg/m^2^)	1.411	1.224–1.626	<0.001
Age (years)	1.016	0.946–1.092	0.655
Male sex	0.594	0.206–1.717	0.336
Multivariable Model 2			
Preserved muscle mass	2.261	0.812–6.291	0.118
BMI (kg/m^2^)	1.422	1.225–1.651	<0.001
MNA-SF score	0.999	0.811–1.229	0.990
Multivariable Model 3			
Preserved muscle mass	2.160	0.764–6.108	0.147
BMI (kg/m^2^)	1.449	1.238–1.697	<0.001
MNA-SF score	0.970	0.785–1.199	0.781
Number of medications	0.988	0.815–1.199	0.904

**Abbreviations:** MNA-SF, Mini Nutritional Assessment–Short Form. **Footnote:** Model 1 included age, sex, BMI, and muscle mass status. Model 2 included muscle mass status, BMI, and the MNA-SF score. Model 3 included muscle mass status, BMI, the MNA-SF score, and the number of medications. Models 2 and 3 were alternative parsimonious specifications adopted because of the limited number of outcome events and were not nested within Model 1; the coefficients of every variable entered into each model are presented. Analyses involving muscle mass status were restricted to complete cases, and the participant with a missing bioelectrical impedance measurement was excluded; the corresponding sample sizes were 112 for Model 1, 110 for Model 2, and 109 for Model 3. Age- and sex-adjusted sensitivity analyses for Models 2 and 3 are presented in Supplementary Table S1. The outcome variable was FR = 1 (calf circumference larger than the finger ring). This category was considered a negative test result for low muscle mass; therefore, odds ratios (ORs) > 1 indicate an increased likelihood of a negative finger-ring test result (FR = 1). For the variable preserved muscle mass, the reference category was low muscle mass.

**Table 5:** Discriminative Performance (AUC) of Combined Models for Identifying Low Muscle Mass and Results of Likelihood-Ratio Tests.

Model	AUC	LR Test *p*-Value
Finger-ring test alone	0.643	—
Finger-ring test + MNA-SF	0.674	0.127
Finger-ring test + calf circumference	0.701	0.019
Finger-ring test + age + sex	0.737	0.004
Finger-ring test + MNA-SF + age + sex	0.737	0.009
Full model	0.752 (optimism-corrected: 0.710)	0.008

**Abbreviations:** AUC, area under the receiver operating characteristic curve; LR, likelihood ratio; MNA-SF, Mini Nutritional Assessment–Short Form. **Footnote:** The outcome variable was low muscle mass. Each combined model was compared with the finger-ring test–only model using a likelihood-ratio (LR) test. Because the models were nested, the DeLong test was not used to compare differences in ROC curve areas; therefore, only AUC values and LR test *p*-values are presented. For the full model, the apparent AUC was 0.752, whereas the optimism-corrected AUC estimated using 1000 bootstrap resamples was 0.710. Model calibration and performance were acceptable (Hosmer–Lemeshow test *p* = 0.155, Nagelkerke R^2^ = 0.254, classification accuracy 68.2%). The finger-ring test provided significant incremental information beyond age and sex (LR test *p* = 0.001), whereas its incremental contribution beyond BMI was not statistically significant (LR test *p* = 0.138).

## Data Availability

The data supporting the findings of this study are available from the corresponding author upon reasonable request.

## Supplementary Materials

The following supporting information can be downloaded at: https://www.mdpi.com/article/10.3390/medicina62081447/s1. Table S1: Age- and sex-adjusted sensitivity analyses for Models 2 and 3 of Table 4.
