# Supplementary material for: Finger-Ring Test and Low Muscle Mass in Older Adults with Chronic Kidney Disease: A Cross-Sectional Study
Source: Medicina (Kaunas). 2026 Jul 25;62(8):1447. doi: 10.3390/medicina62081447 (PMC13514527; doi:10.3390/medicina62081447)
Supplement: Supplementary file 1 [file medicina-62-01447-s001.zip › medicina-4414629-supplementary.pdf]

Supplementary Table S1. Age- and sex-adjusted sensitivity analyses for Models 2 and 3 of Table 4.

Model 2 (nutritional model, age- and sex-adjusted; n = 110)

Variable	OR	95% CI	p-value
Age (years)	1.025	0.951–1.104	0.522
Male sex	0.503	0.162–1.564	0.235
BMI (kg/m^2)	1.383	1.187–1.612	<0.001
Preserved muscle mass	2.600	0.886–7.629	0.082
MNA-SF score	1.048	0.836–1.314	0.683

Model 3 (extended nutritional model, age- and sex-adjusted; n = 109)

Variable	OR	95% CI	p-value
Age (years)	1.032	0.957–1.113	0.409
Male sex	0.478	0.150–1.519	0.211
BMI (kg/m^2)	1.404	1.193–1.654	<0.001
Preserved muscle mass	2.526	0.847–7.533	0.097
MNA-SF score	1.024	0.814–1.287	0.842
Number of medications	1.004	0.823–1.225	0.968

Abbreviations: BMI, body mass index; CI, confidence interval; MNA-SF, Mini Nutritional Assessment–Short Form; OR, odds ratio.

Footnote: The outcome variable was FR=1 (calf circumference larger than the finger ring), considered a negative finger-ring test result. For *preserved muscle mass*, the reference category was *low muscle mass*. Analyses were restricted to complete cases. These models correspond to Models 2 and 3 of Table 4 with age and sex added; the number of events per variable was 10.0 and 8.2, respectively, which is why they are presented as sensitivity analyses rather than as primary models. BMI remained the only statistically significant independent determinant in both models.
